# Should additional value elements be included in cost-effectiveness analysis in pharmacoeconomic evaluation: a novel commentary

**DOI:** 10.1186/s12962-023-00490-4

**Published:** 2023-10-28

**Authors:** Lihua Sun, Shiqi Li, Xiaochen Peng

**Affiliations:** 1 School of Business Administration, Shenyang Pharmaceutical University, Shenyang, Liaoning China; 2 Shanghai Health Development Research Centre, Shanghai, 201199 China

## Abstract

In recent years, international academics recognized that quality-adjusted life-years (QALYs) may not always fully capture the benefits produced by an intervention, and considered incorporating additional elements of value into cost-effectiveness analysis (CEA). Examples of these elements are adherence-improving factors, insurance value, value of hope, and real option value, which form the “value flower”. In order to explore whether it is scientific and reasonable to incorporate additional elements into CEA, this paper focuses on what pharmacoeconomic evaluation should do and what it can do. By elaborating the connotation of value, the connotation of decision, and tracing the origin of pharmacoeconomic evaluation, we believe that it is unscientific and unreasonable to incorporate additional elements of value into CEA, which has exceeded the essential connotation and capability of pharmacoeconomic evaluation. The analysis results belong to the theoretical level, empirical test is needed to verify the correctness and scientificity of this conclusion in the future.

## Introduction

**Fig. 1:**
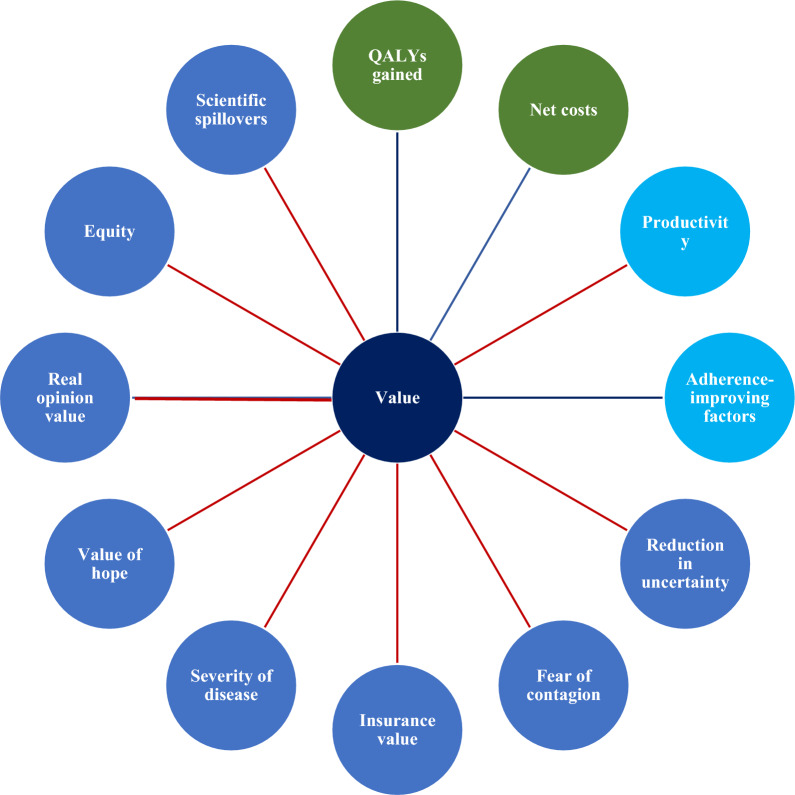
Elements of value. Note. Green circles: core elements of value; light blue circles: common but inconsistently used elements of value; dark blue circles: potential novel elements of value; blue line: value element included in traditional payer or health plan perspective; and red line: value element also included in societal perspective

With the rapid expansion of aging populations and rising medical expenditures all over the world, the importance of optimal allocation and efficient utilization of limited healthcare resources has become more and more prominent [1]. Pharmacoeconomics (PE) evaluation is now gaining prominence given it provided valuable evidence for drug resource allocation decisions. Cost-effectiveness analysis (CEA) is the most commonly used method in PE evaluation, of which the underlying principle is to compare the additional costs to the health gains, typically expressed as quality-adjusted life-years (QALY) when assessing the cost-effectiveness of some existing alternative treatment(s). QALYs and costs often form the basis of value assessments based on CEA, labeled as cost-utility analysis (CUA). Despite capturing the key driver of health gain in terms of length and quality of life, many scholars in recent years think that QALY has limitations. This metric may not always fully capture the health (or well-being) of patients, or incorporate individual or community preferences about the weight to be given to health gain [2]. For example, some believe [3] that disease severity reflects the value of a drug to some extent. Compared with mild diseases, the public will prefer to priortize the resources on the severe ones, therefore, the severity of a disease should be considered in CEA. The value of a treatment that can prolong life but cannot cure a disease should be recognized, for the reason that it can bring the possibility of adopting meidcal technology in the future, which is defined as “Real option value” [4]. Later, ISPOR summarized and published the additional elements that could be incorporated into CEA, such as productivity, adherence-improving factors, insurance value, value of hope, and real option value (collectively referred to as "additional value elements"), which have formed the “value flower” [5] (Fig. 1).

Since proposing the idea of adding additional elements, many researchers have launched a fierce debate on it. Various scholars have different views on elements that QALY should cover. For example, some scholars believe that including the value element of "productivity improvement" in QALY will lead to the occurrence of inequality problems, because different groups have differences in labor value [6]. Chinese scholar Shanlian Hu believes that China needs to combine the macro level of value in "people-centered" with the micro level of value in "patient-centered". The right to health is a basic right of the Chinese people, and the authorities should provide basic medical and healthcare services to the people, especially implement the medical security system to alleviate the poverty due to illness. The people have the obligation to jointly participate in the construction of China's basic medical and healthcare system, also have the right to basic medical and healthcare security. Hence, such considerations should be taken into account in CEA as additional value elements either [7]. Further research flourishes the "value flower", and focus of debate has become to explore how to quantify these additional value elements to account for the benefits of CEA.

Although considering more "additional value elements" is a new trend, but we have some different critical views on this. Considering that quantifying these additional elements is a complicated task that has numerous challenges and requires a lot of limited social resources, it may be more important and necessary to study whether it is scientific and reasonable to incorporate additional value elements into CEA of PE evaluation adopting dialectical thinking. This study traces the origin of PE evaluation, and conducts theoretical analysis based on what Pharmacoeconomics should do and what can be done objectively, so as to provides a valuable perspective into the development of PE evaluation.

## Body

### What pharmacoeconomic evaluation should do

#### Theoretical analysis

In view of the objective existence of scarcity of resources, technical economics and engineering economics that take economic evaluation as the core have already developed into a relatively mature scientific theory and technology for decision-making in the 1960s, and have been widely used in many countries, industries and fields. However, it was not until the mid-1960s that economic problems in the field of medicine and healthcare were investigated as the study of “Estimating the Cost of Illness” [8] conducted in the context of the rapid increase in medical demand and the sharp rise in medical expenditure, and then pharmacoeconomics was born in the late 1980s [9]. Pharmacoeconomics is the science of studying the economic problems and rules of drug resource utilization in the pharmaceutical field, and how to improve the allocation and utilization efficiency of drug resources, so as to achieve the maximum improvement of health status with limited drug resources [1]. PE evaluation is the most basic content of Pharmacoeconomics research. Economic evaluation theories and methods in other fields are the basis for the birth and development of PE evaluation. The difference between PE evaluation and economic evaluation in other fields is mainly presented in the identification and measurement of costs and benefits. However, the role of PE evaluation is consistent with the role of economic evaluation in other fields—that is, not decision-making, but to provide one basis for decision-making. Thus, what PE evaluation should do is the provision of economic evidence for the selection of drug-related clinical intervention programs in the healthcare system.

PE evaluation is conducted to identify, measure, and compare the costs and consequences (i.e., clinical, economic, and humanistic) of pharmaceutical products and services [1]. The term “cost” is well understood, that is, the monetary representation of the resources consumed. The term “consequences” is used to describe the results and value of pharmaceutical interventions, and traditionally refers to “clinical (health) gains”, and its connotation depends on the goal pursued by clinical pharmacotherapy. The ultimate goal of clinical pharmacotherapy is to achieve healthy longevity [10]. which refers to the greatest goal of drug use is the realization of “rational drug use”, defined as “patients receive medications appropriate to their clinical needs, in doses that meet their own individual requirements for an adequate period of time, at the lowest cost to them and their community”. Simply put, it is to ensure that medication is safe, effective, economical, and appropriate [11]. Inevitably, the goal of “rational drug use” is the foundation of any healthcare policy-making, therefore, PE evaluation contains the evaluation of safety, effectiveness and cost of the interventions, which serves as the sub-objective of the economical goal. For the factors that are hardly to quantify, quantitative description should be used to reflect the differences, which is commonly used in economic evaluation in other fields. Thus, the core connotation of “clinical (health) gains” in PE evaluation is the clinical safety and effectiveness.

#### Dialectical analysis of additional value elements

Based on theoretical analysis, the core connotation of clinical gains (safety and effectiveness) in the PE evaluation should specifically determine whether CEA incorporates the additional value elements in the “value flower”. Only factors that juxtapositional to safety and effectiveness should be taken into account, meanwhile they are not necessarily required to quantify as part of the "value". For example, the adherence-improving factors, the clinical gains might increase in case of improving the patient adherence to treatments. This relationship implies that these two factors belong to a cause-and-effect relationship. Adherence-improving is the “cause”, and health outcomes (safety and efficacy) of interventions are the “effect”. In addition, health gains improvement will increase the productivity of patients in a causal relationship framework. The “cause” is the health outcomes of interventions, the “effect” is the improvement of productivity. Therefore, the adherence-improving and improvement of productivity are already embedded in the CEA and should no longer be listed as additional value elements. In other words, medication compliance as an additional value element will lead to double counting.

These analyses propose the question of whether the CEA should include the additional elements of value in the “value flower” that are causally unrelated to the core connotation of the clinical gains of PE evaluation. On the rationality of goals side, what PE evaluation should do is the provision of economic evidence for realizing of rational use of drugs. Nonetheless, the additional elements of value in “value flower”, such as fear of contagion, value of hope and severity of disease have exceeded the connotation covered by the economical goal, as well as the goal of “rational drug use (Or that rational drug use goals don't address issues like equity)”. Hence, these factors all go beyond the scope of PE evaluation, and CEA should ignore such additional value elements.

Moreover, some of the additional value elements are difficult to quantify, the measurement of elements such as equity, value of hope, real-option value lacks the universal accepted quantitative research techniques [3, 11]. Though methods based on questionnaire survey have been used to evaluate the elements such as insurance value and fear of contagion, a divergence of opinion still exists [3]. The inclusion of the additional value elements that immaturely quantified in addition to the core ones will reduce the accuracy of the measurement of “clinical (health) gains” and complicate the quantitative analysis in CEA.

### What pharmacoeconomic evaluation can do

#### Theoretical analysis

The most popular application of PE evaluation is to support decision-makers in making rational decisions regarding pharmaceuticals [1]. Nonetheless, PE evaluation is not decision-making. Decision-making is a completely dynamic process, which not only refers to the selection of alternatives, but also refers to the determination of objectives (a desirable outcome to be achieved in a decision), formulation, selection, and implementation of plans until the realization of goals [12].

**Fig. 2:**
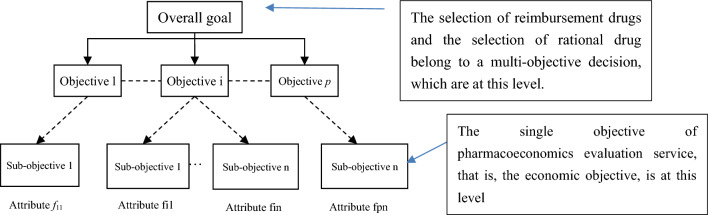
Multi-objectives hierarch

According to the number of objectives that need to be addressed in the decision, decision-making can be divided into single- and multi-objective decisions [13, 14]. PE evaluation (CEA) provides the basis for the single objective decision of economy. The optimal solution of the single-objective decision problem is one and only, for example, the optimal solution of the single-objective decision with the goal of “economical” is the economical option. However, the solution of a multi-objective decision problem is not unique, and it is usually impossible to obtain the absolute optimal solution for each objective at the same time because multi- and single-objective decisions consider different value measures in the decision-making process. In the multi-objective decision, the highest hierarchy of objectives (fundamental objectives [14, 15]) is what we ultimately care about in the decision, which is often not very specific and not easy for being calculated. Subobjectives (means objectives [14, 15]) describe how we achieve our fundamental objectives, which are more specific than fundamental objectives, and are easier to quantify. Value measures are the final tier of objectives hierarchy (Fig. 2). A value measure is a scale to assess how much we attain an objective, which has many synonyms, such as attribute, performance, factor, level, and characteristic [15]. They are all scales that can directly or indirectly evaluate the degree of realizing a specific goal.

#### Dialectical analysis of additional value elements

Based on the above-mentioned characteristics of decision-making, it is not difficult to find that the decision-making goal directly supported by PE evaluation is the economic goal in rational drug use, which belongs to a single-objective decision. That is, what PE evaluation can do is to support the single-objective decision. Under the goal, PE evaluation can help decision-makers select the most economic one among multiple alternatives, and any decision-maker will make the same optimal choice under this criterion.

The selection of reimbursement drugs and the selection of rational drug belong to a multi-objective decision, and economy is only a sub-objective of its many goals. For example, the decision of rational drugs needs to consider safety, effectiveness, economy, and appropriateness of clinical use of drugs, so the most economical one is not necessarily the final choice. Therefore, the weight of each subobjective will change in the multi-objective decision due to the preferences and specific requirements of decision-makers. Hence, in a multi-objective decision, PE evaluation is a value measures tool of economic subobjective (The CEA of PE evaluation belongs to the narrow category of micro level, rather than the economic concept of macro level). Additional elements of value in "value flower" belong to value measures of different sub-objectives in the multi-objective decision (multiple micro targets). Of course, PE evaluation tools cannot, cover them all. If one insists on incorporating additional elements of value into CEA, the essence of PE evaluation is no longer a tool to provide an economic basis for decision-making, but has been transformed into a decision-making tool due to the intention of including every aspect of value into the equation of CEA, where a decision will be made directly based on the comparison of the ICER (Incremental cost-effectiveness ratio) and the threshold.

## Conclusion

In conclusion, in terms of what should be done or what can be done for PE evaluation, it is unscientific and unreasonable to incorporate additional elements of value in “value flowers” into CEA, which has exceeded the essential connotation and capability of PE evaluation.

## Data Availability

Not applicable.
